# Multi-omic profiling of breast tumor microenvironment uncovers a role of mitochondrial calcium gatekeepers

**DOI:** 10.7150/jca.95979

**Published:** 2024-05-13

**Authors:** Yen-Dun Tony Tzeng, Pei-Yi Chu, Su-Boon Yong, Tzu-Sheng Hsu, Ling-Ming Tseng, Ming-Feng Hou, Jim Jinn-Chyuan Sheu, Jui-Hu Hsiao, Chia-Jung Li

**Affiliations:** 1 Institute of Biomedical Sciences, National Sun Yat-sen University, Kaohsiung 804, Taiwan.; 2 Department of Surgery, Kaohsiung Veterans General Hospital, Kaohsiung 813, Taiwan.; 3 Department of Pathology, Show Chwan Memorial Hospital, Changhua 500, Taiwan.; 4 Department of Allergy and Immunology, China Medical University Children's Hospital, Taichung 404, Taiwan.; 5 Research Center for Allergy, Immunology, and Microbiome, China Medical University Hospital, Taichung 404, Taiwan.; 6 Institute of Molecular & Cellular Biology, National Tsing Hua University, Hsinchu 300, Taiwan.; 7 Comprehensive Breast Health Center, Taipei Veterans General Hospital, Taipei 112, Taiwan.; 8 Division of Breast Surgery, Department of Surgery, Center for Cancer Research, Kaohsiung Medical University Chung-Ho Memorial Hospital, Kaohsiung 807, Taiwan.; 9 Institute of Clinical Medicine, National Cheng Kung University, Tainan 704, Taiwan.; 10 Department of Surgery, Kaohsiung Municipal Minsheng Hospital, Kaohsiung 802, Taiwan.; 11 Department of Obstetrics and Gynecology, Kaohsiung Veterans General Hospital, Kaohsiung 813, Taiwan.; 12 Institute of BioPharmaceutical Sciences, National Sun Yat-sen University, Kaohsiung 804, Taiwan.

**Keywords:** Breast cancer, Spatial transcriptomic, MiCU, Single-cell RNA-Sequence, Precision medicine

## Abstract

In this study, we aimed to elucidate the role of mitochondrial calcium uptake 1/2 (MiCU1/2) in breast cancer (BRCA) by employing a comprehensive multi-omics approach. Unlike previous research, we utilized a novel web application tailored for whole tumor tissue, single-cell, and spatial transcriptomics analysis to investigate the association between MiCU1/2 and the tumor immune microenvironment (TIME). Our gene set enrichment analysis (GSEA) provided insights into the primary biological effects of MiCU1/2, while our CRISPR-based drug screening repository identified potential effective drugs. Our study revealed that high MiCU1/2 expression serves as an independent diagnostic biomarker, correlating with advanced clinical status and indicating poorer recurrence-free survival (RFS) rates in BRCA patients. Additionally, spatial transcriptome analysis highlighted the heightened expression of MiCU1/2 in tumors and its relevance in surrounding immune cells. Furthermore, using the CIBERSORT algorithm, we discovered a positive correlation between MiCU1/2 levels and macrophage infiltration, underscoring their potential impact on immune infiltration. We also identified expression patterns of immune-related genes associated with responses against various immune cell types, including CXCL, MIF, GDF, SPP1, and IL16. Finally, our pharmacogenomic screening identified potential small molecule drugs capable of effectively targeting breast cancer cells with elevated MiCU1/2 expression. Overall, our study establishes MiCU1/2 as a promising novel biomarker for BRCA diagnosis and prognostic prediction, as well as a potential therapeutic target, highlighting the importance of exploring these pathways to advance patient care and outcomes in BRCA treatment.

## Introduction

Recent years have seen rapid advancements in breast cancer research, with multifactorial carcinogenesis and studies elucidating biological and molecular pathways driving tumor progression and drug resistance 1, 2. This has led to the development of various molecular targeted therapies, making breast cancer a prominent example of successful precision medicine. The first step involves novel blood-based diagnostics providing clinically useful information non-invasively. However, there is an urgent need to identify novel biomarkers for early diagnosis, particularly to guide initial treatment and predict recurrence or drug resistance following novel targeted therapies 3, 4.

MiCU1 is pivotal in maintaining mitochondrial calcium balance and acts as a key regulator of calcium ion intake within mitochondria 5, 6. MiCU1 shares a 25% sequence homology with two homologs, the protein products of human genes EFHA1 and EFHA2 7. Both of these proteins possess N-terminal mitochondrial targeting sequences and are detectable in various mouse tissues, denoted as MiCU2 and MiCU3, respectively. However, MiCU3 is presently excluded from the list due to its lack of specific mitochondrial localization 8. Conversely, MiCU2 exhibits a distinct mitochondrial localization pattern similar to MiCU1, featuring a highly conserved EF-hand structural domain. MiCU2 interacts with both MiCU1 and MCU, and akin to MiCU1, it is presumed to reside within the intermembrane space of mitochondria rather than in mitochondrial matrix proteins 9. *In vivo* silencing of MiCU2 does not impact mitochondrial membrane potential or mitochondrial respiratory chain function, but it impedes mitochondrial Ca^2+^ uptake. Downregulation of MiCU2 results in decreased mitochondrial Ca^2+^ levels, while its overexpression in MiCU1-silenced HeLa cells restores mitochondrial Ca^2+^ uptake, aligning the phenotype closer to the wild type 10, 11.

While previous studies have established the link between MiCU1/2 cancer, this research aims to comprehensively assess both MiCU1 and MiCU2 in the breast cancer tumor microenvironment, particularly focusing on their influence on immune cell regulation, which remains poorly understood. Additionally, the specific molecular mechanisms underlying the impact of MiCU1/2 on immune cells have yet to be elucidated. To address this gap and uncover the potential drivers of energy metabolism shaping cellular heterogeneity and metastatic features in breast cancer, we examined MiCU1/2 alterations using various approaches including tissue microarray analysis of human BRCA specimens, fresh BRCA biopsies from human biobanks, scRNA-seq, and spatial transcriptomics. Through functional enrichment analysis, we highlighted the involvement of MiCU1/2 in immune responses, allowing for a deeper understanding of their roles in shaping the tumor microenvironment and interactions with different immune cell types. Notably, our immunological assessment revealed a positive correlation between MiCU1/2 expression and increased macrophage infiltration, suggesting a potential role for MiCU1/2 in regulating macrophages within the tumor microenvironment. These insights hold promise for developing strategies to counteract immunosuppression and hinder tumor progression.

## Materials and methods

### Patient samples

A set of 25 paired breast tumor and non-tumor tissues were procured from breast cancer patients enrolled in the Kaohsiung Veterans General Hospital Human Biobank. Written informed consent was acquired from all participants, and the study was sanctioned by the Ethical Governing Committee, adhering to pertinent guidelines and regulations (Approval Code: KSVGH22-003).

### Multi-omics analysis

Multi-omics analysis involved leveraging various bioinformatics resources. Gene Expression Profiling Interactive Analysis 2 (GEPIA2) serves as an interactive platform widely used for visualizing gene expression profiles by integrating The Cancer Genome Atlas (TCGA) and The Genotype-Tissue Expression (GTEx) data. Within the Tissue Samples tab, GEPIA2 offers insights into tumor vs. normal comparisons, encompassing 60,498 gene types and 198,619 isoforms. Our study utilized GEPIA2 to examine the expression levels of the hub gene across different cancer types. Furthermore, UALCAN provides access to graphs and plots depicting gene expression and survival curves, along with the ability to evaluate promoter hub gene information and conduct pan-cancer gene expression analysis 12. We utilized UALCAN to explore patient survival data across various cancer types based on hubgene expression. Additionally, cBioCancer was employed to investigate TCGA DNA variations within the MiCU1/2 genes, pinpointing their precise genomic locations. Furthermore, we examined the correlation between gene expression and multiple clinical variables using data from cBioCancer. Moreover, we utilized DriverDBv4, a comprehensive cancer omics database incorporating somatic mutation, RNA expression, miRNA expression, protein expression, methylation, copy number variation, and clinical data, along with annotation bases 13, 14.

### Protein level analysis

Protein level analysis and immunohistochemistry involved investigating MiCU1/2 expression using the Human Protein Atlas (HPA) database for human tumor and normal tissue samples. Tissue microarray (TMA) slides from SuperBioChips Laboratories, Seoul, Korea, containing breast cancer, metastatic cancer, and normal tissue specimens, were analyzed. The characteristics of all the patients included in this study are listed in Supplementary Table 1. Immunohistochemistry (IHC) utilized a biotin-free immune enzymatic antigen detection kit (BioTnA, TAHC01D, Kaohsiung, Taiwan) with specific antibodies to label target proteins. Samples were fixed with paraformaldehyde, permeabilized with Triton X-100, and blocked with 5% BSA to reduce non-specific binding. Primary antibodies were incubated overnight at 4°C, followed by secondary antibody incubation at room temperature for 30 minutes. TMA slides were examined and evaluated by Li-Tzung Pathology Laboratory, Kaohsiung, Taiwan, using a BX61VS® microscope (Olympus, Tokyo, Japan) 15. The H-score statistical method assesses both the staining intensity and the percentage of positive cells in a sample. Staining intensity is categorized into four levels: level 0 indicating no expression, level 1 for weak expression, level 2 for moderate expression, and level 3 for strong expression. The total score, ranging from 0 to 300, is calculated by multiplying the staining intensity by the percentage of positively labeled cells.

### Processing of spatial transcription data

Our analysis utilized previously published spatial transcription (ST) data (STDS0000049). We aggregated unique molecular identifiers (UMIs) within each spot defined by bin100 and annotated clusters based on hematoxylin and eosin (H&E) sections. Spots containing more than 10% mitochondrial genes or fewer than 200 detected gene counts were excluded. Further annotation was conducted using cell markers. The resulting dataset was analyzed and visualized to assess gene expression and spatial distribution. To reduce dimensionality, we employed the RunPCA function followed by the FindNeighbors and FindClusters functions to cluster similar spatial transcriptome points. This approach enabled us to estimate the prevalence of common cell types based on the average representation matrix of various clusters, which is particularly suitable for spatial transcriptome analysis 16.

### Evaluation of the tumor immune cell microenvironment

The application of single-cell RNA sequencing (scRNA-seq) has emerged as a potent technique for exploring cellular diversity and gene expression at the individual cell level. In our investigation, scRNA-seq data sourced from the GEO repository underwent rigorous quality control (QC) using the R package Seurat. This rigorous QC process ensures the selection of high-quality cells while minimizing variations associated with different experimental batches. Through the utilization of "BiocManager" and the Gene Set Variation Analysis (GSVA) package in R, we identified distinct cell subpopulations via unified manifold approximation and projection (UMAP) clustering. Annotation of these cell types involved comparing their expression patterns with established cell marker genes using the "SingleR" package in R. Furthermore, we investigated the relationship between gene expression and genetic markers associated with tumor-infiltrating immune cells. To accomplish this, we employed the TIMER2 database and CIBERSORT method to assess the correlation between gene expression and immune infiltration. Correlations between gene expression and cell abundance (e.g., CD4+ T cells, CD8+ T cells, neutrophils, macrophages, eosinophils, and natural killer cells) were calculated using the Pearson correlation coefficient. Additionally, we utilized ESTIMATE to evaluate the correlation between gene expression and ESTIMATE score, immune score, and stromal score 16.

### Batch effect removal

Batch effects, which often occur in datasets containing multiple donors or samples, introduce considerable variability among the samples. To evaluate these effects, we utilized a metric that considers both information entropy and Euclidean distance within the UMAP graph for each dataset. Higher information entropy values indicate more uniform batch mixing. The entropy calculation formula is provided.



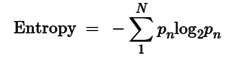



In detail, when assessing batch effects, we observed that most datasets were affected, leading to substantial variation across samples. To mitigate these effects, we employed a metric based on information entropy and Euclidean distance within the UMAP graph. A higher entropy value suggests a more balanced mixing of batches. If the ratio of the maximum entropy to the median entropy exceeded 4, we implemented conventional correlation analysis (CCA) using Seurat v4.0.4 to mitigate batch effects.

### Statistical analysis

Statistical analysis was conducted using the R software (ver. 4.0.2). The results are presented as mean ± standard deviation (SD), and statistically significant differences were defined as those with p < 0.05.

## Results

### Genetic variations and functional analysis of MiCU family members in breast cancer

**Figure 1:**
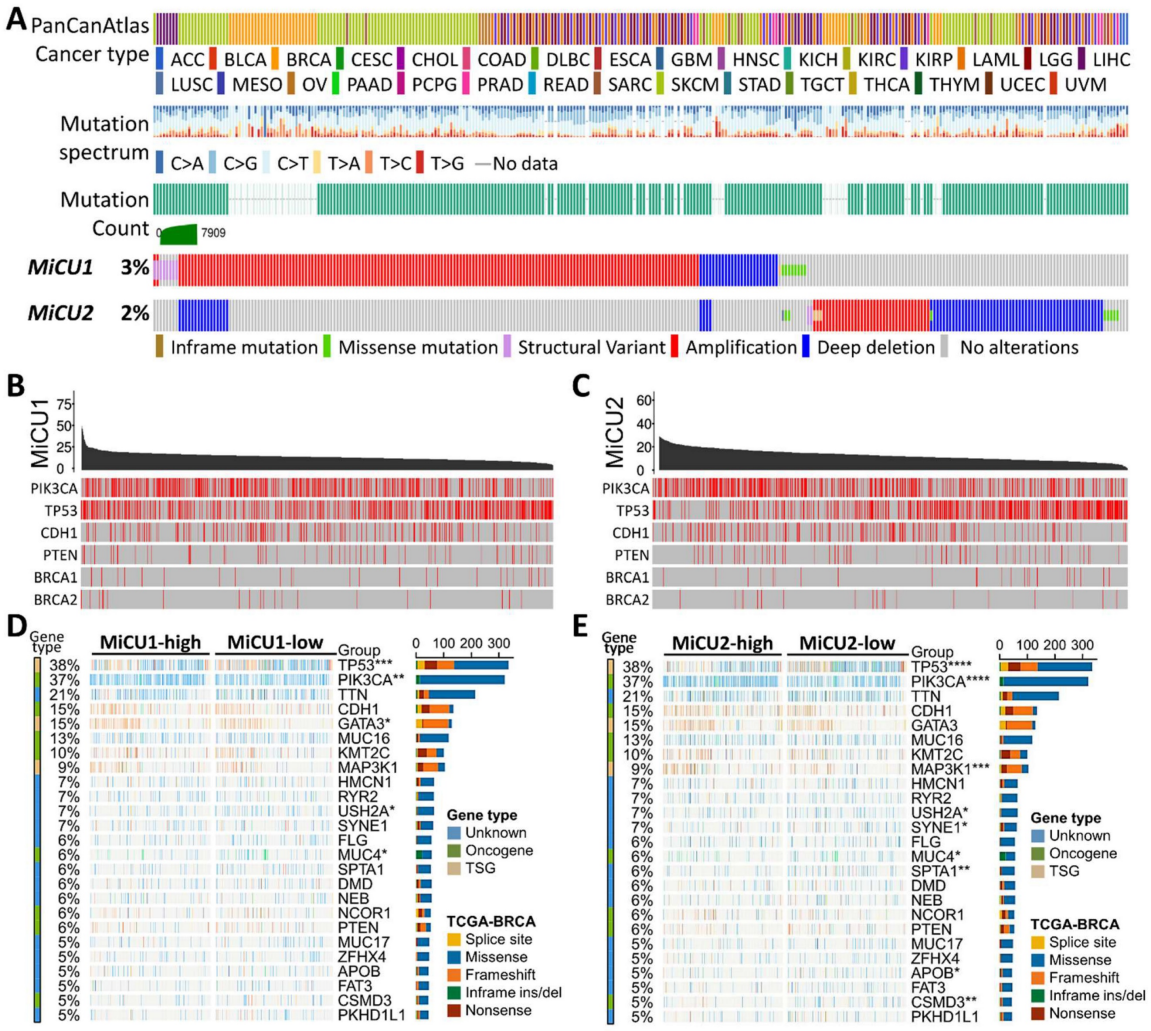
In-depth examination of the frequency and distribution of mutations in MiCU1/2 genes across breast cancer. (A) Scrutinizing mutations across various cancer types unveils the spectrum of genetic alterations impacting MiCU1/2 genes. (B & C) Exploring the interplay among highly mutated genes in MiCU1/2 elucidates potential interactions and co-occurrences, with identified mutation sites facilitating detailed analysis. (D & E) Fisher's exact test contrasts mutation frequencies across different MiCU1/2 expression levels, providing insights into mutation distribution within each group.

In our investigation, we delved into the genetic alterations present in individual members of the MiCU family in breast cancer using the cBioPortal dataset. Our analysis revealed that both MiCU1 and MiCU2 genes exhibited altered landscapes in 3% and 2% of BRCA patients, respectively. The most common types of mRNA alterations observed in the MiCU family were amplification and deep deletion (Fig. 1A). Additionally, we examined the association between MiCU1/2 mutations and other well-known cancer-related genes such as PIK3CA, TP53, and CDH1 (Fig. 1B&C). Furthermore, utilizing data from TCGA database, we depicted MiCU1/2 expression patterns in BRCA patients through a waterfall plot, highlighting the top 25 affected genes (Fig. 1D&E). This comprehensive analysis shed light on the diverse scenarios involving MiCU1/2.

**Figure 2:**
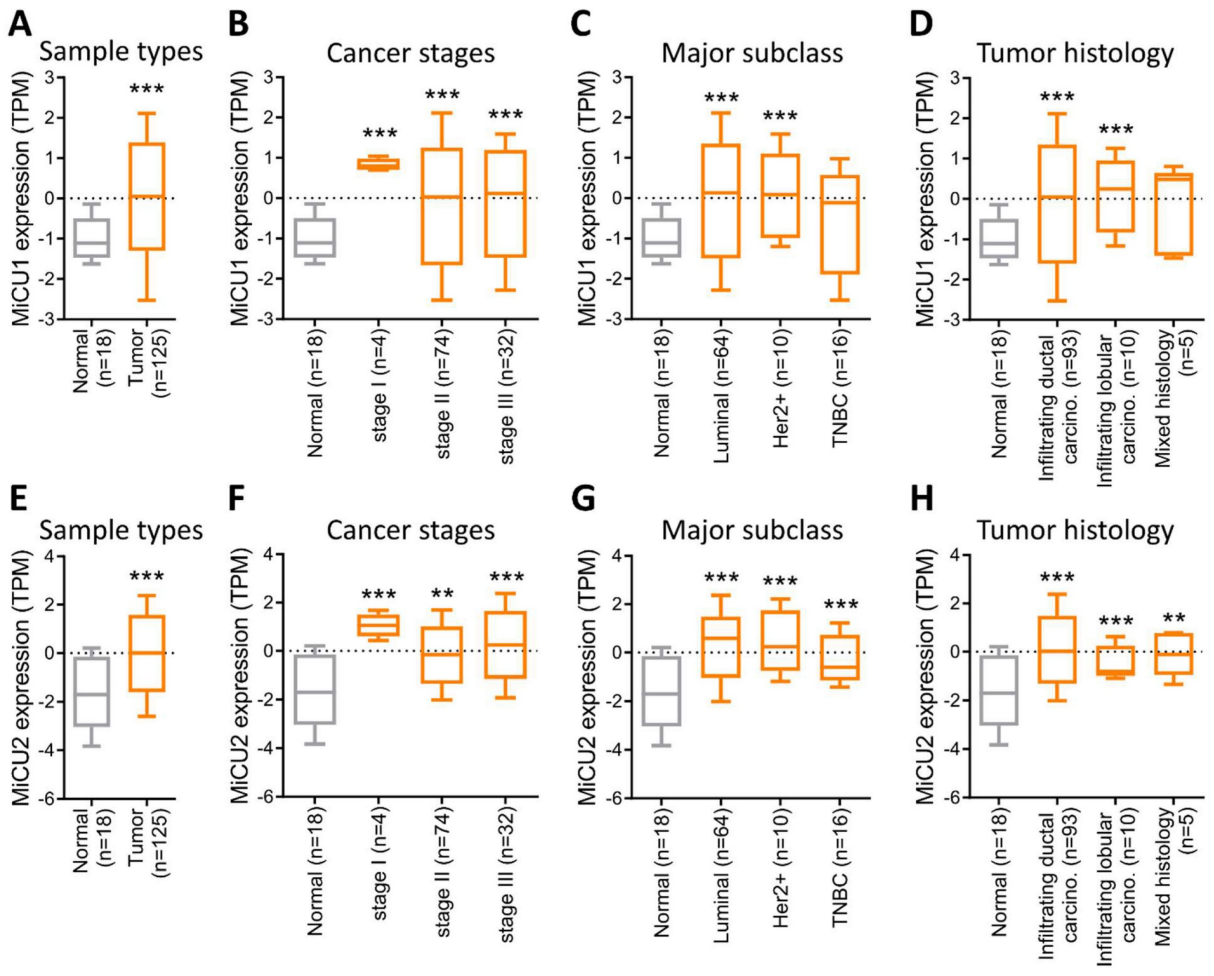
Comparison of MiCU1/2 gene expression in BRCA. (A & E) Expression levels of MiCU1/2 were assessed in BRCA tumor and non-tumor tissues, depicted by box plots representing expression across various stages of BRCA (B & F), major subclasses (C & G), and tumor histology (D & H). ** P < 0.01, *** P < 0.001.

Subsequently, we utilized data from the National Cancer Institute's Clinical Proteomic Tumor Analysis Consortium (CPTAC), a database aimed at enhancing our understanding of cancer's molecular underpinnings through extensive proteomic and genomic analyses. This dataset allowed us to assess the expression of MiCU1/2 across various contexts in breast cancer, encompassing different cancer types, stages, major subtypes, and tumor histology. Our analysis revealed robust expression of MiCU1/2 in breast cancer overall (Fig. 2A&E), with particularly elevated levels observed in late-stage disease (Fig. 2B&F). Notably, while MiCU1 displayed heightened expression in luminal and Her2+ subtypes, no significant difference was noted in triple-negative breast cancer (TNBC), contrasting with MiCU2. Furthermore, MiCU1 exhibited abundant expression in infiltrating ductal and lobular carcinoma histological types (Fig. 2C&G), whereas MiCU2 showed significant differences between normal and other histological types (Fig. 2D&H).

### Elevated MiCU1/2 levels correlate with the progression of clinical breast cancer

**Figure 3:**
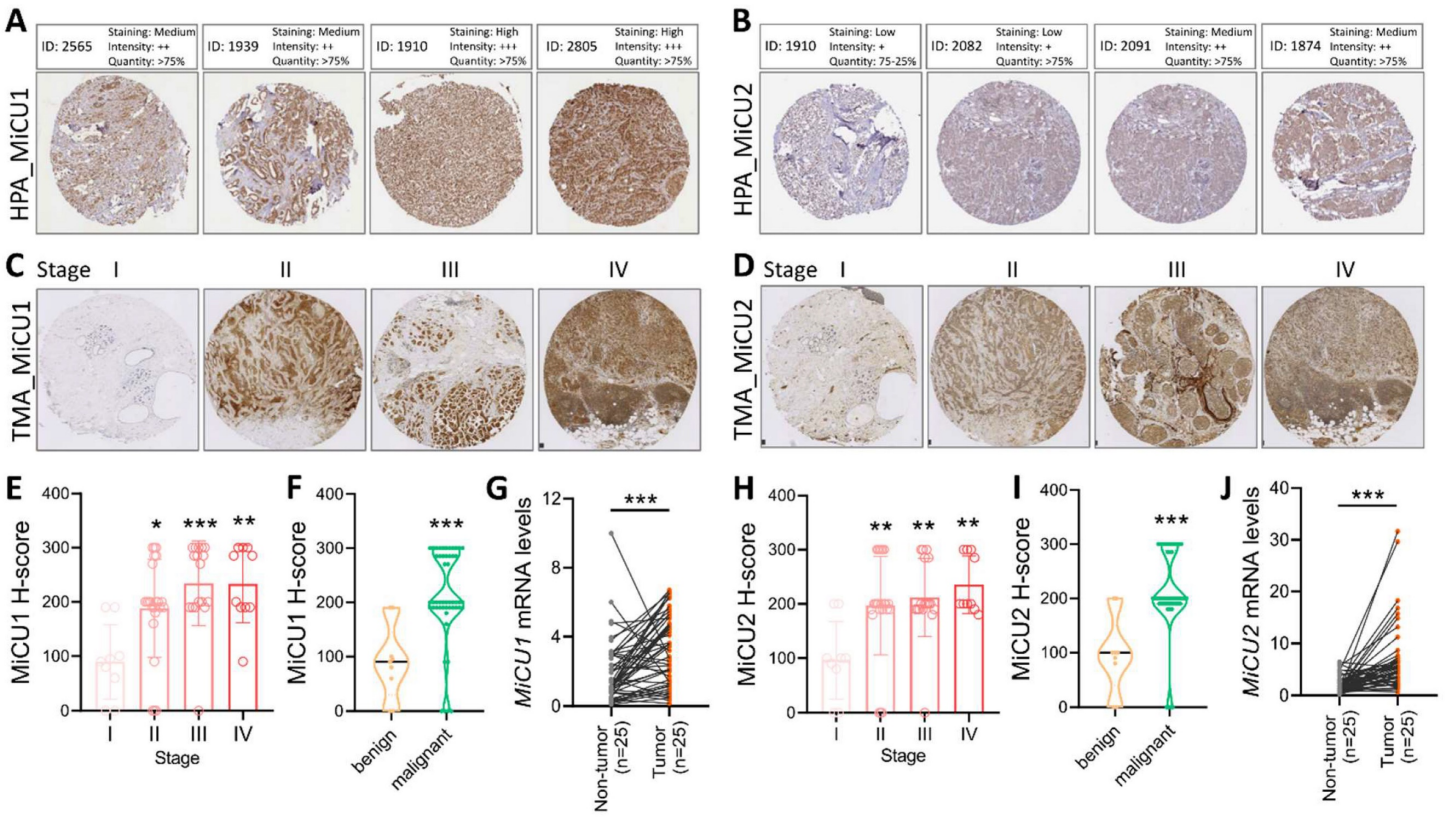
The examines MiCU1/2 alterations in breast cancer. (A & B) Protein expression levels in breast cancer tissues are evaluated using immunohistochemistry from the Human Protein Atlas. (C & D) Representative images display staining intensities. (E & H) Expression across cancer stages is shown in box plots. (F & I) Violin plots illustrate expression in benign and malignant tissues. (G & J) qPCR analysis is conducted on paired samples from human biobank. * P < 0.05, ** P < 0.01, *** P < 0.001.

In this study, we examined the levels of MiCU1/2 proteins in breast cancer using data sourced from the HPA database. We analyzed protein levels in biopsies from four randomly selected patients, revealing a substantial increase in MiCU1 protein levels across various breast cancer samples (Fig. 3A), while MiCU2 expression showed a lower abundance compared to MiCU1 (Fig. 3B). To validate these findings from public databases, we conducted immunohistochemistry (IHC) analysis of tumor tissues utilizing a breast cancer tissue microarray (TMA to assess MiCU1/2 expression patterns (Fig. 3C &D). The results of IHC staining demonstrated a significant rise in MiCU1/2 expression levels with advancing breast cancer stages. Evaluation of the IHC scores by clinical pathologists and subsequent H-score analysis indicated a prominent expression of MiCU1/2 starting from the second stage of breast cancer (Fig. 3E&H). Additionally, consistent results showed abundant expression of MiCU1/2 in both benign and malignant tumors (Fig. 3F&I). Furthermore, we randomly selected 25 pairs of breast tumor and non-tumor tissues from the KSVGH biobank to analyze the mRNA expression of MiCU1/2 in these biopsies. The results from quantitative PCR (qPCR) demonstrated a significant upregulation of MiCU1/2 in breast cancer tissues compared to non-tumor tissues (Fig. 3G&J).

### Examining the MiCU1/2 transcriptome with a single-cell RNA sequencing and spatial transcriptomics

**Figure 4:**
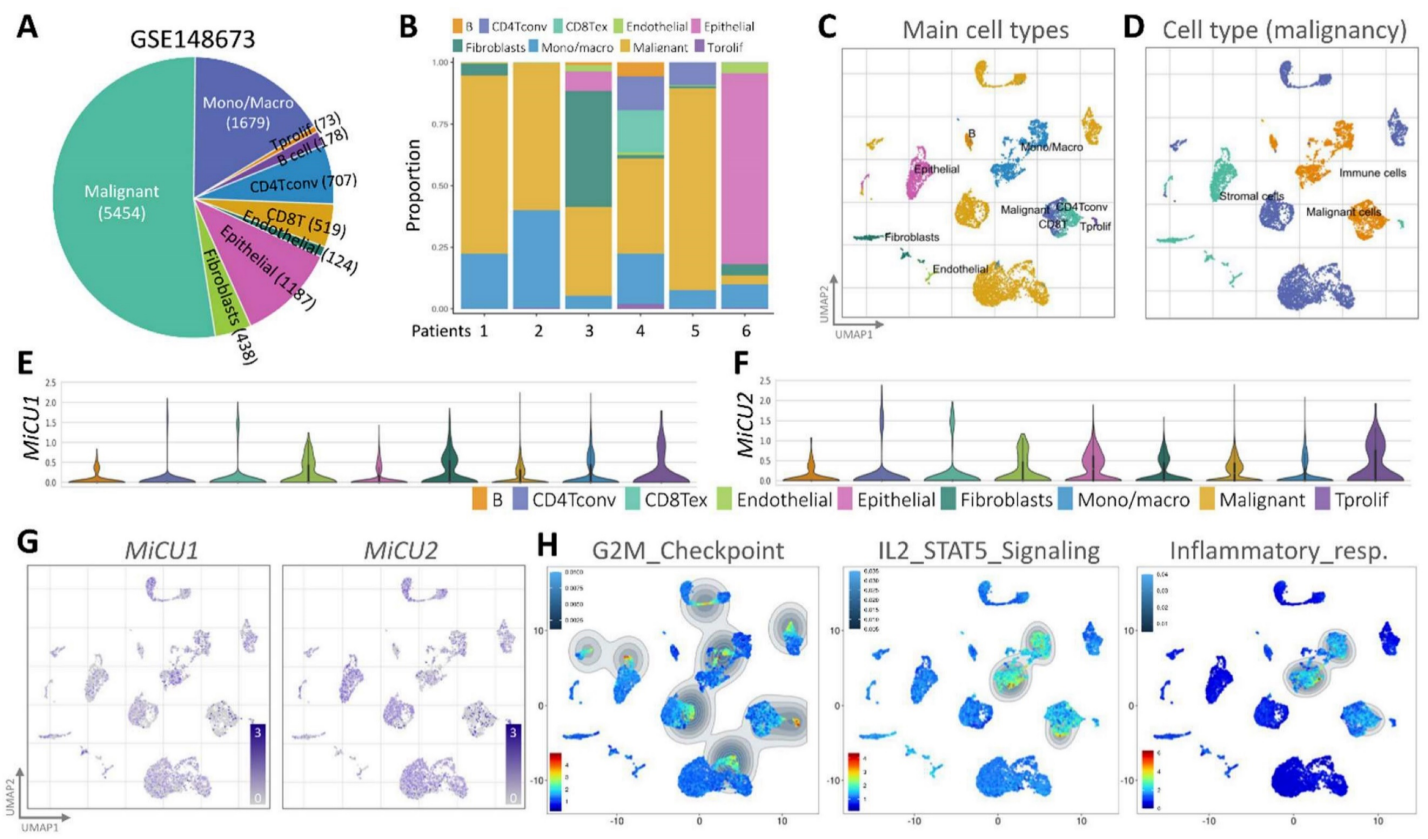
Analyzing immune cell populations through single-cell RNA sequencing. (A and B) Utilizing single-cell RNA-sequencing analysis, the distribution of immune cell populations is illustrated in the public dataset and the GSE148673 dataset, respectively. (C and D) Utilizing the UMAP technique, BRCA cells are visually represented, with colors indicating predominant cell type and malignancy status. (E & G) Expression levels of MiCU1/2 across different cell clusters are depicted using violin and UMAP plots. (H) Results of GSEA for G2M checkpoint, IL2/STAT5 signaling, and inflammatory response signaling pathways in gene expression clusters.

To examine the transcriptome of MiCU1/2 at the breast cancer single-cell level and study the diversity of cell types in the breast cancer microenvironment, we performed analyzes using an existing breast cancer single-cell RNA sequencing database (GSE148673), half of which tumor cells, and the remainder were other immune cell populations (Fig. 4A). This database covers 9 different cell types and is derived from human breast cancer patient samples (Fig. 4B). We used UMAP diagrams to identify these 9 different key cell groups and divided them into mail cell types and cell types (Fig. 4C-D). Using the most differentially expressed genes in each cluster, we identified cell type-specific markers that are critical for subsequent cell type classification (Fig. 4E-F). After carefully examining the expression and distribution of MiCU1/2 in these single-cell RNA sequencing databases, we then used Gene Set Enrichment Analysis (GSEA) to analyze the relationship between MiCU1/2 and G2M checkpoint, IL2/STAT5 signaling, and Inflammatory response. MiCU1/2 expression increased in the corresponding region (Fig. 4G-H).

**Figure 5:**
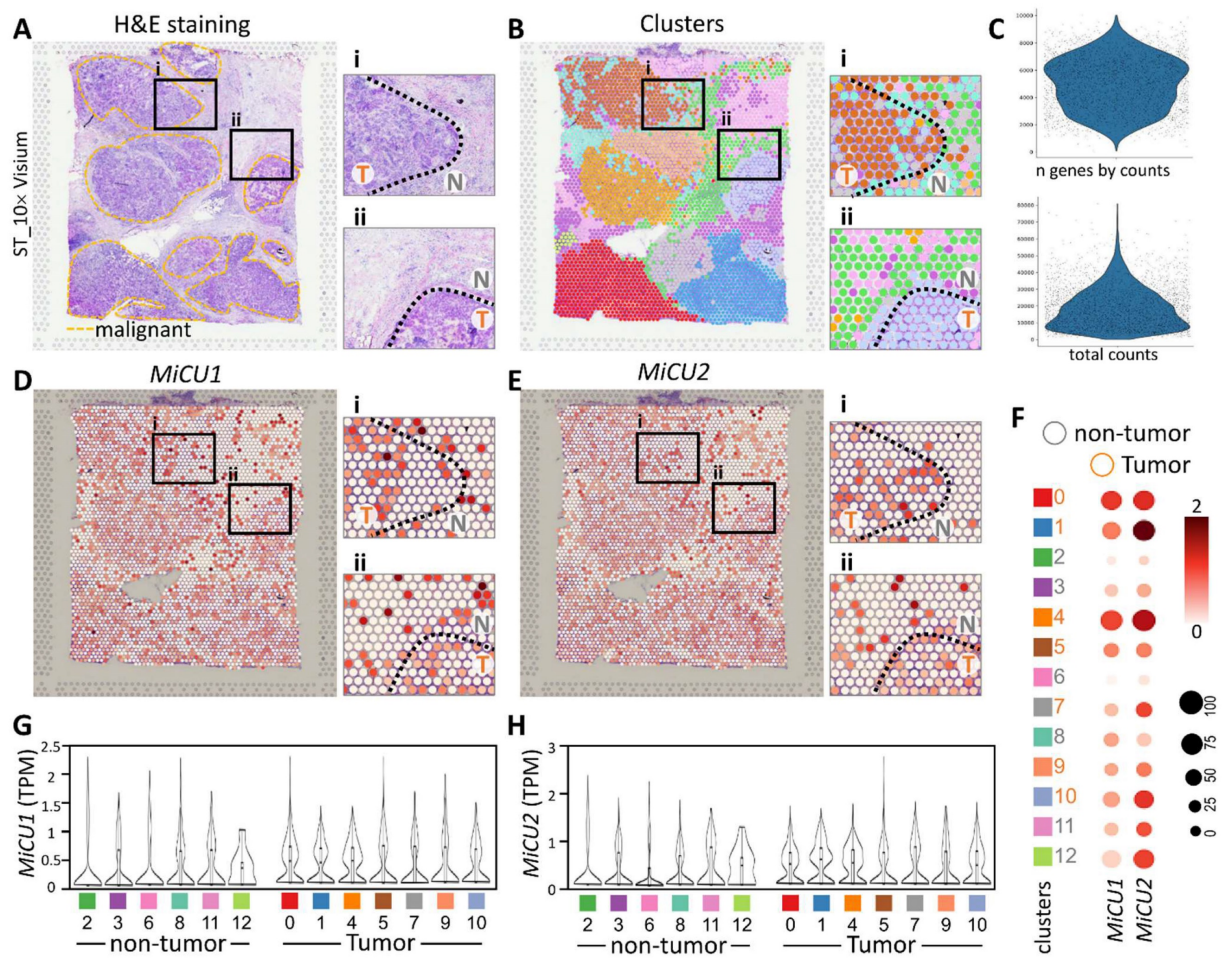
Spatial transcriptomics revealed that MiCU1/2 reprogramed the tumor microenvironment distribution. (A-B) Spatial transcriptomics enabled the analysis of tissue sections, facilitating the identification and precise alignment of clusters with morphological features observed through hematoxylin and eosin staining and cluster mapping. Malignant regions are highlighted by yellow dashed outlines, with magnified images showing genetic variants in boxes i and ii. (C) Key characteristics of the dataset, including total counts and gene counts, are provided. (D) Utilizing 10× Visium spatial gene expression, the spatial distribution and genetic alterations involving MiCU1/2 in distinct tissue sections were examined. (F) Dot plots illustrate the expression levels of various genes across different clusters. (G-H) Violin plots depict the transcript levels of MiCU1/2 in both tumor and non-tumor regions. T: tumor, N: non-tumor.

In this investigation, our focus was on the ST data obtained from breast cancer patients, as documented in a previously published study on breast cancer (STDS0000026). Initially, we annotated clusters based on hematoxylin and eosin (H&E) sections and cell markers, allowing us to pinpoint regions exhibiting distinct tumor morphology (Fig. 5A). Notably, the cellular disparities between non-tumor and tumor sections are prominently evident in the magnified image. Subsequently, employing unsupervised clustering, we organized similar ST points into 12 distinct clusters (Fig. 5B). Utilizing gene counts and total counts, we constructed violin plots to visualize the distribution of gene expression. Following data normalization, we identified the top 33,538 highly variable genes in each sample (Fig. 5C). Figure 5D illustrates the transcript levels of MiCU1/2 across different regions and cell types within this spatial transcriptome. The findings underscore the efficacy of unsupervised clustering analysis in grouping ST points with akin characteristics into coherent clusters. The dot plot further delineates the levels of MiCU1/2 in non-tumor and tumor regions (Fig. 5F). Moreover, Figure 5G & H presents the outcomes of unsupervised clustering analysis depicting the distribution and expression patterns of these two genes across diverse cell clusters.

### Intercellular communication network inference reveals breast tumor microenvironment

**Figure 6:**
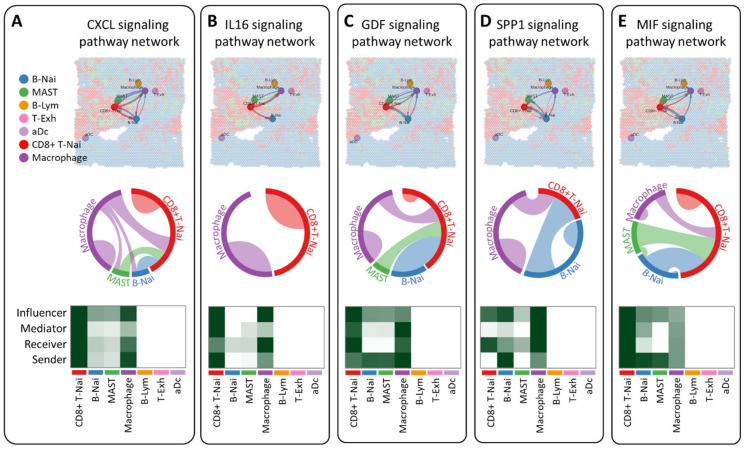
Investigation of intercellular communication networks among diverse cell types using CellChat, employing spatial transcriptomics to discern distinct signaling pathways and network centrality scores for the CXCL (A), IL16 (B), GDF (C), SPP1 (D) and MIF (E) signaling pathway across various cell types.

CellChat employs pattern recognition techniques to quantitatively assess ligand-receptor interactions, predicting crucial incoming and outgoing signals for specific cell types. In the CXCL, IL16, GDF, and SPP1 signaling pathway network, seven cell types are identified as potential targets, including CD8+ T-Nai, B-Nai, MAST, macrophage, B-Lym, T-Exh, and aDc. Notably, CD8+ T-Nai and macrophage clusters exhibit robust signaling as both influencers and senders. While CD8+ T-Nai, B-Nai, MAST, and macrophage cells exhibit signaling activities, B-Lym, T-Exh, and aDc show no signals. Each cell type serves as both a secretory cell, releasing various cytokines or ligands, and a target cell, receiving signals through receptors targeted by ligands from the same or other cell types (Figure 6A-D). The ligand-receptor-mediated communication between different cell types is likely implicated in breast cancer development. Interestingly, macrophage migration inhibitory factor (MIF) signals exhibit weak activity across all roles of influencer, mediator, receiver, and sender (Fig. 6E).

**Figure 7:**
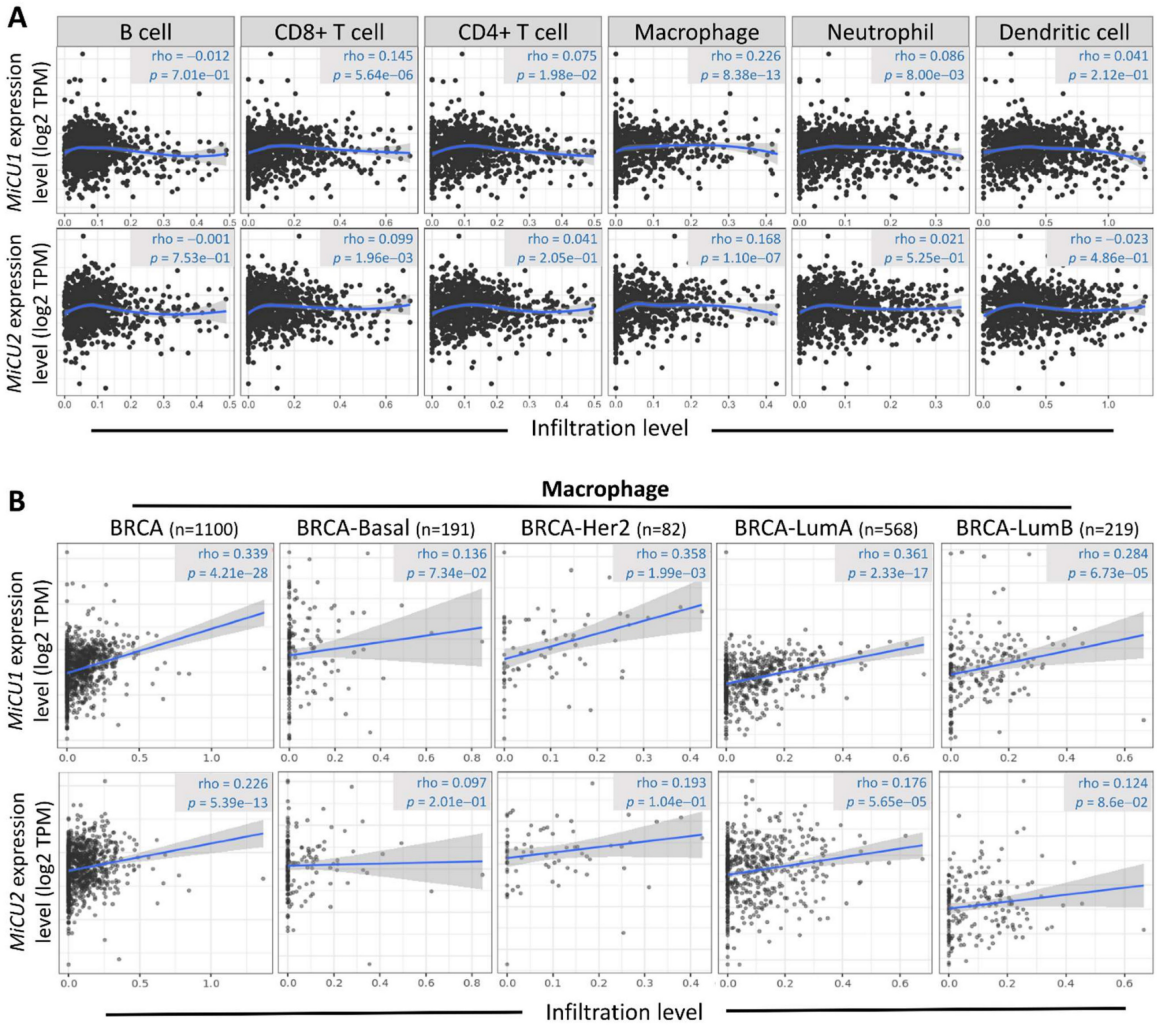
Association between the level of MiCU1/2 mRNA expressed and the infiltration of immune cells. (A) Investigating the relationship between MiCU1/2 expression and the infiltration of B cells, T cells, macrophages, neutrophils, and dendritic cells in TCGA breast cancer samples. (B) Assessing the correlation between MiCU1/2 expression and macrophage infiltration across various subtypes of breast cancer.

The initiation and progression of BRCA are intricately linked to the immune microenvironment, prompting speculation about the potential association between MiCU1/2 and this microenvironment. To delve into this relationship and elucidate its underlying mechanisms, we turned to the TIMER2.0 database. Our analysis revealed a significant correlation between MiCU1/2 and various immune cell types, including T cells CD4+, T cells CD8+, B cells, neutrophils, macrophages, and bone marrow dendritic cells (Fig. 7A). Notably, macrophages exhibit substantial enrichment and infiltration within tumor tissues exhibiting high MiCU1/2 expression levels. Furthermore, in different subtypes of BRCA tissues characterized by elevated MiCU1/2 expression, macrophages demonstrate a positive correlation with MiCU1/2 levels (Fig. 7B). These findings strongly suggest that MiCU1/2 may play a role in promoting the initiation and progression of BRCA through modulation of immune cell infiltration.

### Exploration of small molecule drugs via pharmacogenomics

**Figure 8:**
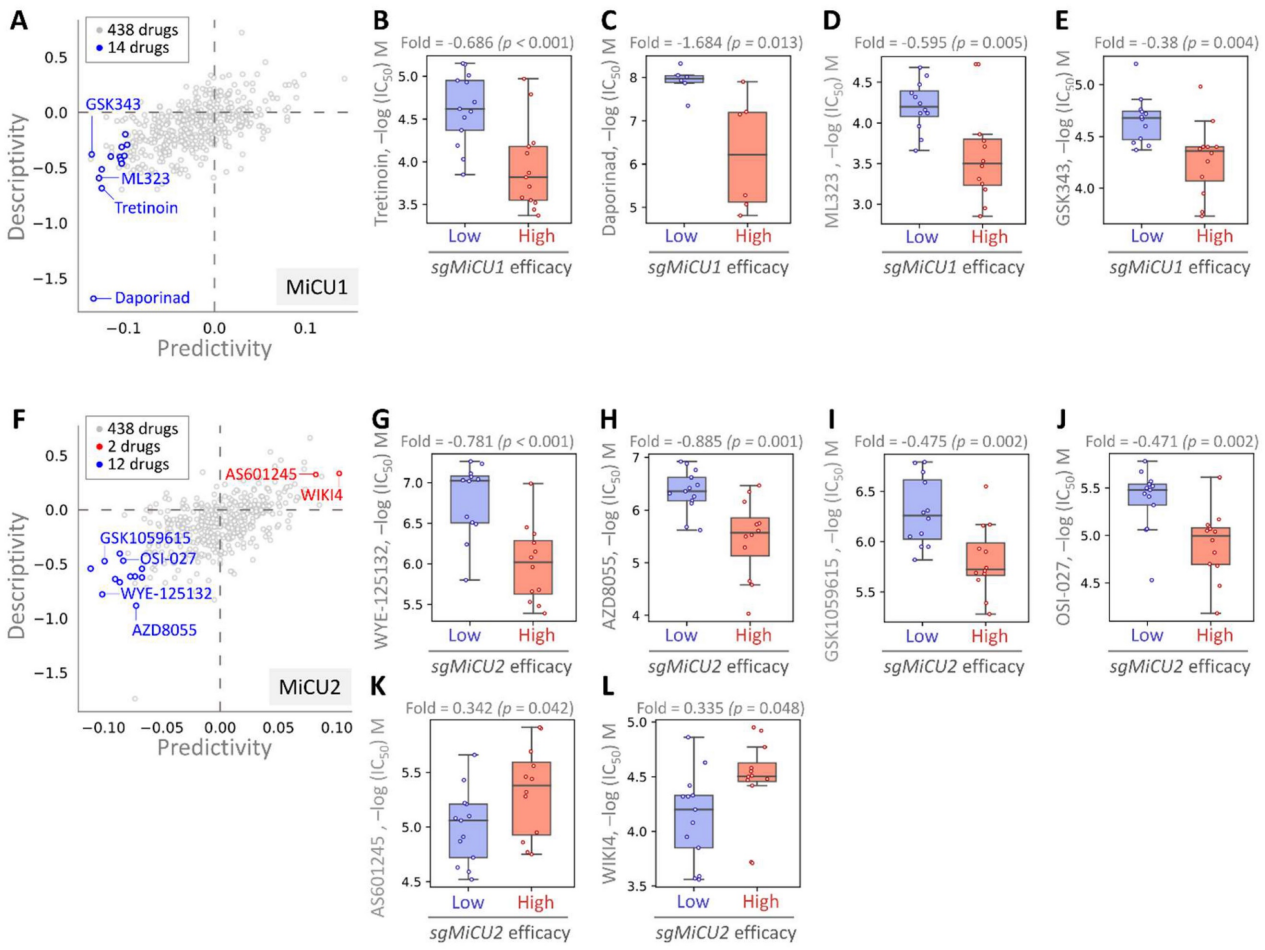
Assessment of drug responsiveness and pharmacogenomic profiling in breast cancer cells. (A-F) Utilizing pharmacogenetic databases to screen for genetic signatures and identify potential drug candidates. Predictability is measured by assessing the variation in CRISPR efficacy between cells exhibiting high and low responses to the target drug. Evaluating the drug sensitivity of sgMiCU1 (B-E) and sgMiCU2 (G-L) gene knockout BRCA cell lines to a range of small molecules. Boxplots depict the maximum inhibitory concentrations of various small molecule drugs for gene knockout.

This investigation aimed to uncover potential drugs effective against BRCA by leveraging the Drug Genome Database to identify compounds exhibiting enhanced efficacy in the presence of elevated MiCU1/2 expression. Through cross-correlation analyses between drug responses and CRISPR knockdown of BRCA using single-guide RNA (sgRNA) across diverse BRCA cell lines, we dissected the impacts of 438 drugs on sgRNA-mediated MiCU1 in BRCA cells individually (Fig. 8A). This analysis unveiled 14 small molecule drugs, with the top four demonstrating heightened inhibitory effects: Tretinoin, Daporinad, ML323, and GSK343, exhibiting altered potency (Fig. 8B-E). Furthermore, in the case of MiCU2, we screened 438 drugs, identifying 12 with inhibitory effects and 2 that can promote MiCU2 expression in breast cancer cells (Fig. 8F). Among these, four small molecule drugs displayed notable inhibitory effects: WYE-125132, AZD8055, GSK1059615, and OSI-027. Conversely, AS601245 and WIKI4 were found to promote MiCU2 expression in breast cancer cells (Fig. 8G-L). Overall, these findings suggest the potential utility of these drugs as anticancer agents targeting MiCU1/2 to modulate breast cancer cell proliferation.

## Discussion

Changes in MiCU1 expression are a common feature across various cancers, with elevated MiCU1 levels correlating positively with unfavorable clinical outcomes, particularly in ovarian cancer 17. In hepatocellular carcinoma (HCC), studies have demonstrated that patients exhibiting high expression of MCU/MiCU2 have a poorer prognosis in terms of overall survival (OS). Additionally, increased expression of both MCU and MiCU1 has been statistically linked to worse survival outcomes, with higher clinical stage and poorly differentiated histological grade 18. In colorectal adenocarcinoma (COAD), the expression of MCUR1 and MiCU2 escalates with disease progression, and elevated MICU2 levels are significantly associated with reduced OS 19. Prior studies have revealed that RPS3 regulates melanoma cell proliferation and apoptosis primarily by modulating Ca^2+^ signaling and MiCU1-dependent mitochondrial pathways. Knocking out the RPS3 gene suppresses MiCU1 expression, leading to mitochondrial calcium overload 20. In head and neck squamous cell carcinoma, MiCU1 expression is elevated, and inhibiting MiCU1 disrupts mitochondrial calcium homeostasis and membrane potential stability, thereby inhibiting apoptosis and reducing Bcl-2 expression 21. Manipulating MiCU1 expression enhances the growth and proliferation of renal clear cell carcinoma cells, whereas increasing MiCU1 levels markedly suppresses cell growth and proliferation. These findings imply a close association between MiCU1 and MiCU2 the onset and progression of renal clear cell carcinoma. Nevertheless, the precise mechanism by which MiCU1 modulates cell proliferation in renal clear cell carcinoma remains elusive, and alterations in MiCU1 expression may be linked to abnormal expression of forkhead box D1 (FOXD1) in renal clear cell carcinoma 22, 23.

Currently, our understanding of the upstream regulatory mechanisms governing MiCU1/2 expression remains limited, although several studies have suggested the involvement of miRNAs in regulating MiCU1/2. For instance, previous research has unveiled the role of the LHFPL3-AS1/miR-580-3p/STAT3 axis in promoting melanoma progression through the activation of the JAK2/STAT3 pathway 24. In ovarian cancer, MiCU1 has been implicated in conferring resistance to chemotherapy and facilitating glycolysis, contributing to tumor progression 25. Furthermore, microRNA-195 has been identified as a key regulator of ovarian cancer cell growth by modulating MiCU1 expression levels 17. Additionally, circular RNA hsa_circ_0072309 has been shown to inhibit the progression of non-small cell lung cancer by targeting miR-580-3p 26. These findings underscore the complex regulatory network involving MiCU1/2 and highlight their significance in various cancer types.

The TIME is crucial for supporting tumor growth and progression, influencing various aspects such as metastasis and response to treatment 27. The composition and functional status of different immune cell subpopulations within TIME are fundamental in these processes. In this study, we conducted a comprehensive analysis to explore the relationship between the expression of MiCU1/2 and the infiltration of diverse immune cells into tumors 28, 29. Our findings revealed a strong positive correlation between the expression of MiCU1/2 and macrophages, suggesting a potential role of MiCU1/2 in regulating the differentiation and activation of immune cells within TIME. More than half of the cellular constituents within tumor tissue are comprised of macrophages 30. Initially thought to eliminate tumor cells, it has been discovered through research that macrophages can coexist with tumor cells and may even facilitate tumor advancement and metastasis 31. Tumor-associated macrophages (TAMs), particularly the M2 phenotype, are known to contribute to tumor progression by promoting angiogenesis, enhancing cancer stem cell properties, and inhibiting the activity of cytotoxic T lymphocytes (CTLs) through the secretion of immunosuppressive cytokines such as IL-10 and TGF-β. We observed that MiCU1/2 positively regulates TAM-mediated tumor invasion, indicating a possible positive feedback loop between them. This suggests that MiCU1/2 may contribute to the immunosuppressive effects within the tumor microenvironment by promoting TAM activity, thereby facilitating tumor immune evasion.

Current evidence suggests that MiCU1/2 plays a crucial role in tumor formation and may contribute to tumor progression. Analysis of various databases including TCGA, GTEx, GEO, and HPA revealed significantly elevated expression of MiCU1/2 in BRCA, which correlated with poor prognosis and reduced survival rates among patients. We also confirmed the expression of MiCU1/2 in breast cancer through tissue microarray and human biobank samples, demonstrating its independent prognostic impact on BRCA and its correlation with clinical characteristics. However, the specific mechanisms by which MiCU1/2 regulates BRCA progression remain unclear. Tumor progression and immunomodulation are closely intertwined, suggesting a potential role for MiCU1/2 in BRCA pathogenesis through the immune microenvironment. Subsequent analysis revealed a positive correlation between MiCU1/2 expression and immune cell infiltration levels, particularly increased levels of macrophages. This suggests that elevated MiCU1/2 expression may induce immune suppression by altering immune cell composition and infiltration levels, while also affecting immune checkpoint recognition, allowing BRCA to evade immune surveillance. While our research has shed light on the significance of MiCU1/2 in BRCA, including its impact on the immune microenvironment, growth, and prognosis, there are still gaps in our understanding. Further investigation is needed to elucidate the mechanisms through which MiCU1/2 influences the development of hepatocellular carcinoma cells.

This study concentrates on employing multi-omics approaches to investigate unexplored aspects related to MiCU1 and MiCU2 in breast cancer research. Firstly, our study employed a novel multi-omics approach utilizing a web application tailored for whole tumor tissue, single-cell, and spatial transcriptomics analysis, which represents a significant advancement in the field. This approach allowed us to comprehensively assess the diagnostic and prognostic significance of MiCU1/2 in breast cancer (BRCA) patients. The utilization of spatial transcriptome analysis revealed heightened expression of MiCU1/2 in tumors and its relevance in surrounding immune cells, shedding light on the intricate interplay between mitochondrial calcium handling and the tumor immune microenvironment (TIME). Additionally, our study identified a positive correlation between MiCU1/2 levels and macrophage infiltration using the CIBERSORT algorithm, highlighting the potential impact of mitochondrial calcium regulation on immune infiltration dynamics within the tumor microenvironment. Furthermore, the expression patterns of immune-related genes associated with responses against various immune cell types were delineated, providing insights into the immunomodulatory role of MiCU1/2 in BRCA. Finally, pharmacogenomic screening identified potential small molecule drugs capable of targeting breast cancer cells with elevated MiCU1/2 expression effectively, opening up avenues for personalized therapeutic interventions targeting mitochondrial calcium regulation. Overall, these novel findings underscore the potential of MiCU1/2 as a promising biomarker for BRCA diagnosis, prognostic prediction, and therapeutic targeting, highlighting the importance of exploring these pathways to advance patient care and outcomes in BRCA treatment.

## Conclusion

In conclusion, our findings demonstrate that MiCU1/2 is significantly downregulated in BRCA tissues, yet exhibits high diagnostic and prognostic value for BRCA, while also being intricately linked to tumor immune regulatory mechanisms. This evidence underscores the potential of MiCU1/2 as promising targets for cancer immunotherapy and novel immune-related biomarkers for diagnosing and prognosticating BRCA.

## Supplementary Material

Supplementary table.
