# Supplementary material for: Multi-omic profiling of breast tumor microenvironment uncovers a role of mitochondrial calcium gatekeepers
Source: J Cancer. 2024 May 13;15(12):3663–74. doi: 10.7150/jca.95979 (PMC11190767; doi:10.7150/jca.95979)
Supplement: Supplementary file 1 — Supplementary table. [file jcav15p3663s1.pdf]

Supplementary Table 1. The relationship of clinicopathological characteristics with MiCU1 and MiCU2 expression in BRCA patients.

		MCU Expression			FOXP3 Expression			TGFb1 Expression		
**Variables**	**Total**	**Low**	**High**	***p*-Value**	**Low**	**High**	***p*-Value**	**Low**	**High**	***p*-Value**
Case number, n	59	97	143	-	120	120	-	120	120	-
Age	55.0 ± 13.5	53.4 ± 13.2	55.7 ± 13.7	0.36	54.1 ± 13.8	56.3 ± 13.1	0.42	54.1 ± 13.8	56.3 ± 13.1	0.42
Survive, n	240	143	97	0.02	120	120	<0.001	120	120	<0.001
Recurrence, n	194	72	122	0.39	108	86	0.43	108	86	0.43
FIGO stage, n										
I	135	62	73	0.04	94	41	<0.01	94	41	<0.01
II	57	29	28	-	27	30	-	27	30	-
III	26	10	16	-	5	21	-	5	21	-
IV	22	10	12	-	6	16	-	6	16	-
Differentiation, n										
Well	77	41	36	0.03	11	66	<0.01	11	66	<0.01
Moderate/Poor	163	61	102	-	32	131	-	32	131	-
